# Dedicated Representation of Others in the Macaque Frontal Cortex: From Action Monitoring and Prediction to Outcome Evaluation

**DOI:** 10.1093/cercor/bhab253

**Published:** 2021-08-25

**Authors:** Lorenzo Ferrucci, Simon Nougaret, Rossella Falcone, Rossella Cirillo, Francesco Ceccarelli, Aldo Genovesio

**Affiliations:** Department of Physiology and Pharmacology, SAPIENZA, University of Rome, Piazzale Aldo Moro 5, 00185 Rome, Italy; Department of Physiology and Pharmacology, SAPIENZA, University of Rome, Piazzale Aldo Moro 5, 00185 Rome, Italy; Department of Physiology and Pharmacology, SAPIENZA, University of Rome, Piazzale Aldo Moro 5, 00185 Rome, Italy; Institut des Sciences Cognitives Marc Jeannerod, Département de Neuroscience Cognitive, CNRS, UMR 5229, 69500 Bron Cedex, France; Department of Physiology and Pharmacology, SAPIENZA, University of Rome, Piazzale Aldo Moro 5, 00185 Rome, Italy; PhD program in Behavioral Neuroscience, Sapienza University of Rome, 00185 Rome, Italy; Department of Physiology and Pharmacology, SAPIENZA, University of Rome, Piazzale Aldo Moro 5, 00185 Rome, Italy

**Keywords:** action observation, frontal cortex, monkey, outcome, prediction, social

## Abstract

Social neurophysiology has increasingly addressed how several aspects of self and other are distinctly represented in the brain. In social interactions, the self–other distinction is fundamental for discriminating one’s own actions, intentions, and outcomes from those that originate in the external world. In this paper, we review neurophysiological experiments using nonhuman primates that shed light on the importance of the self–other distinction, focusing mainly on the frontal cortex. We start by examining how the findings are impacted by the experimental paradigms that are used, such as the type of social partner or whether a passive or active interaction is required. Next, we describe the 2 sociocognitive systems: mirror and mentalizing. Finally, we discuss how the self–other distinction can occur in different domains to process different aspects of social information: the observation and prediction of others’ actions and the monitoring of others’ rewards.

## Introduction

Social interaction is a fundamental aspect of primates’ lives. Various abilities are necessary for complex social behavior, and many of these are shared between humans and monkeys. Nonhuman primates cooperate (Haroush and Williams 2015; Visco-Comandini et al. 2015), monitor the actions of others (Yoshida et al. 2011; Falcone et al. 2012a; Yoshida et al. 2012), and learn from observation (Subiaul et al. 2004; Chang et al. 2011; Falcone et al. 2012b; Monfardini et al. 2014). Monkeys represent an ideal experimental model because of their evolutionary proximity to humans and their suitability for extracellular recording methods while they are engaged in behavioral tasks.

The ongoing recent development of neural recording technologies (Lebedev and Nicolelis 2017; Mitz et al. 2017; Hong and Lieber 2019) has increasingly overcome the earlier limitations of these methods, providing more data of high temporal and spatial resolution from different cortical and subcortical areas of the brain. Neurophysiological investigations in nonhuman primates offer insight into the neural basis of complex social behaviors that could not be investigated otherwise.

Social interaction requires the ability to differentiate the actions performed by the self from those performed by others, in addition to maintaining shared representations, such as shared goals. Distinguishing between self-intentions and others’ intentions in terms of goals and actions is a prerequisite for predicting and acting appropriately in social situations. When studying goals and actions we should be aware that, in principle, these 2 concepts need to be distinguished, since the same goal can in many cases be achieved through different actions, for example, through either eye or arm movements, or either the right or the left arm could be used during a reaching movement. However, many of the experiments we review here do not differentiate between goals and actions, and the dissociation of goals and actions requires specific experimental paradigms (Saito et al. 2005). We therefore do not make this distinction when reviewing them.

The distinction between self and others is important in our daily lives in many regards, for example, when we interact and coordinate with others in joint actions, which can be defined as “any form of social interaction whereby two or more individuals coordinate their actions in space and time to bring about a change in the environment” (Sebanz et al. 2005). Joint action tasks require individuals to coordinate their actions, and they are a key aspect of many social activities, from moving a couch to playing a duet (Novembre et al. 2012; Ray and Welsh 2018). When acting together, it is important to take into consideration others’ presence by making predictions about their goals and forthcoming actions and anticipating the consequences of those actions.

Another example of the importance of representing others’ actions and their outcomes is social learning. When learning from others, it is essential to keep track of information regarding the consequences of the outcomes of their actions, and who benefits from a reward.

From a clinical point of view, failure of the ability to distinguish between stimuli related to self or originating in the environment has been associated with some of the symptoms of schizophrenia in humans (Blakemore et al. 2000; Peled et al. 2000; Asai et al. 2011). Patients with schizophrenia make errors of agency, attributing external events to themselves, as in the rubber hand illusion (Peled et al. 2000) or perceiving self-produced tactile stimuli as having been externally produced (Blakemore et al. 2000). In individuals with autism spectrum disorder (ASD), failure in false-belief tasks has been associated with a deficit in the process of distinguishing between 2 different perspectives, that is, related to the self or to others (Lombardo and Baron-Cohen 2011). This deficit in human subjects with ASD has been correlated with an “atypical representation” of the self and others in the ventral prefrontal cortex (Lombardo et al. 2010). In a monkey with autistic traits, the neurons in the homologous ventral prefrontal cortex area did not respond to others’ actions (Yoshida et al. 2016).

### Aim of the Review

Our aim is to review the electrophysiology studies that have investigated the presence of dedicated representations of others in primates’ brain. We outline how specific experimental conditions may be necessary to reveal dedicated representation of others, and how the social paradigms adopted in the experiment are critical to interpreting the results.

We suggest that, in the same way as neurons with shared representation form the basis of the mirror system, neurons with dedicated representation underly the ability to infer others’ mental states and form the basis of the mentalizing system.

After describing the 2 main social neural systems, mirror and mentalizing, we focus on electrophysiological studies. For social interactions to be successful, information that comes from multiple domains has to be integrated: we need to monitor the actions of the other to make better predictions and adjust our behavior accordingly. The following sections highlight some studies that provide evidence of the dedicated representation of action monitoring, action prediction, and outcome monitoring during social interactions. We mainly focus on the role of neurons recorded in the macaque frontal cortex.

## Experimental Paradigms for Studying Social Cognition

In the field of social neuroscience, one of the most intriguing challenges is designing specific experimental paradigms to closely reproduce and systematically investigate the wide range of behaviors that characterize social interactions between monkeys in everyday life.

These behaviors, which occur spontaneously in natural environments, must be reproduced in a much less natural setting to be studied in a controlled context. In the growing body of literature on social neurophysiology, the self–other distinction has been studied using experimental paradigms that differ in several respects, and these differences must be taken into account when interpreting the results, because they can critically impact the findings. As we will see in the following sections, the properties of single neurons may vary, and specific classes of cells can be under- or overrepresented, depending on the experimental paradigm used.

To provide critical insights into the self–other distinction, we suggest that 2 main aspects should be considered when choosing an experimental paradigm: the type of “other” and the type of observation.

### Type of “Other”

Research on interactions using nonhuman primates can be based on one of 2 main paradigms: between conspecifics (e.g., monkey–monkey interactive paradigms) or between nonconspecifics (e.g., monkey–human interactive paradigms) (Falcone et al. 2012a; Isoda et al. 2018; Ferrari-Toniolo et al. 2019; Nougaret et al. 2019). Here, we discuss a third possibility as well: interactions with inanimate agents, as reported in some studies as a control for the social aspect of the physical agent.

Monkey–monkey paradigms were first used in behavioral studies to investigate the ability of nonhuman primates to learn by observation (Darby and Riopelle 1959; Myers 1970; Emery et al. 1997; Meunier et al. 2007). Most of the experimental paradigms used in these studies relied on more natural settings, in which the monkeys were relatively free to move and interact with each other naturally. However, monkey–monkey paradigms have also been widely used for behavioral neurophysiology under the constraints that are necessarily involved in laboratory settings. Several studies have used monkey pairs to investigate the neural correlates of various social-cognitive processes, such as observational learning (Isbaine et al. 2015), social facilitation (Demolliens et al. 2017), cooperation (Haroush and Williams 2015), visuomotor coordination (Ferrari-Toniolo et al. 2019), representation of others’ actions (Yoshida et al. 2011), and rewards (Azzi et al. 2012; Chang et al. 2013). Paradigms that explore interactions between conspecifics offer the advantage of being the closest to natural behavior, offering the possibility of investigating behaviors that are particular to monkeys, such as interactions in a competitive context (Fujii et al. 2007; Hosokawa and Watanabe 2012) or between subjects belonging to different social ranks (Santos et al. 2012).

Recently, a growing body of evidence has suggested that monkeys can also successfully interact with nonconspecific agents, such as humans. Observational learning has been investigated in behavioral studies in which monkeys interacted with humans and observed their actions, learned from them, and then acted accordingly (Kuroshima et al. 2008; Falcone et al. 2012b; Monfardini et al. 2014). Monkeys are also able to follow the human gaze and discriminate between human attentional states (Flombaum and Santos 2005; Canteloup et al. 2015).

Monkey–human paradigms began to be adopted in neurophysiological experiments as well, starting with studies that investigated the mirror neuron system (di Pellegrino et al. 1992; Rizzolatti et al. 1996) and initially involved observation only, rather than interaction between agents. In these pioneering experiments, typically, the monkeys’ neural activity was recorded while they observed various types of grasping action performed by the experimenter. More recently, a series of studies investigated monkey–human interactions in an experimental paradigm in which the monkey had to actively monitor the choices of its human partner, with the roles of actor and observer alternating between trials (Falcone et al. 2012a, 2016, 2017; Cirillo et al. 2018). Interactions with a human partner rather than another monkey offer the advantage that the human agent’s behavior can be experimentally controlled. The human agent can act according to a precise behavioral protocol; for example, they can consistently make correct or incorrect choices or perform specific action sequences, based on the experimental question. In this way, it is possible to enable the monkey to predict the human’s future actions and thus to study the neural substrate of this prediction in the monkey’s brain. This substrate is hard to investigate using a monkey–monkey paradigm because of uncertainties regarding the behavior of the observed monkey. On the other hand, this approach may seem less natural and the neural representation of the behavior of a human agent may involve different brain areas and neural populations than those that form the representation of a conspecific’s behavior. Moreover, this approach is limited in that it cannot be used to investigate interactions based on the hierarchical structure of the monkey colony or on hierarchies among conspecifics.

Finally, in some studies, monkeys were required to interact with an inanimate agent rather than an animate agent such as another monkey or a human partner. In these studies, the inanimate agent has been either a computer performing choices automatically or a cursor moving on the screen (Subiaul et al. 2004; Ferrucci et al. 2019; Sacchetti et al. 2021). The few neurophysiological experiments thus far that have recorded neural activity during the observation of a cursor rather than the movement of an animate agent have failed to find a unique neural substrate for the “other’s” actions (Cisek and Kalaska 2004; Tkach et al. 2007). The comparison of monkey behavior and neural activity during interactions with animate or inanimate agents could thus shed light on the nature of the processes underlying social interaction and how distinct the neural substrates for self and others are when interacting with different types of agents. We should consider the possibility that the type of social agent may strongly affect the results. Some evidence indicates that the activity of neurons in the lateral prefrontal cortex (lPFC) is modulated by the animacy of the interactive agent (Hosokawa and Watanabe 2012). In other words, neuronal activity differs depending on whether the actor monkey is interacting with another monkey or an inanimate computer agent. Such evidence indicates that, whenever possible, it is important to compare the effects of interacting with animate and inanimate agents using the same experimental paradigm. To promote a dedicated neural representation of others may require the monkey to perceive the other as a separate entity with an independent will, different from its own, and capable of making its own decisions—an aspect that may be lacking during interactions with an inanimate agent. The attribution of animacy has been linked, indeed, to the ability displayed by the observed agent, to possess a mental capacity that confers a certain degree of intentionality (Tremoulet and Feldman 2006). It is possible that the critical difference is not between an animate or inanimate agent per se, but in how the agent is perceived by the observer, whether animate or not. It is important, however, to bear in mind that, when it comes to human subjects, it is possible to manipulate the perceived animacy of something that is inanimate per se (such as an object moving on a screen; we will discuss this point in the next section), whereas in the case of monkeys the physical presence might be an essential prerequisite for the attribution of animacy. This possibility could be addressed for example using robot agents in future studies, to mimic the movement of a real physical agent, in contrast to a moving cursor on a screen. Furthermore, using 2 different observational conditions can help to dissociate what at first sight might appear as a specific other-related signal from a signal that could instead merely represent the inaction of the actor during the observation. If so, using an inanimate control agent may help to clarify the nature of this representation, showing that other’s related signals may actually not be specific to others but can be generalized to any observation condition which requires not to move.

### Type of Observation

In the majority of tasks designed to study the distinction between self and others, the observation condition has been compared with the individual execution condition. However, there is a fundamental difference between these 2 types of observation. In “passive” observation, the animal observes another agent executing an action without the need to extract information to ensure the success of its own future behavior. This was the case in the initial studies on the mirror system (di Pellegrino et al. 1992; Gallese et al. 1996; Rizzolatti et al. 1996). In contrast, in “active” observation the monkey monitors the other’s action, tracks the other’s choices, extracts information from such observations, and acts accordingly, either by taking turns or acting simultaneously. This was the case, for example, in the studies of Falcone et al. (2012a) and Ferrucci et al. (2019), in which the target chosen by the partner in trial *n* defines the target that should be chosen by the actor in trial *n* + 1. The involvement of the actor, and the actor’s ability to extract information from the other agent’s behavior, is then measured behaviorally based on the correctness of the actor’s choices.

Active observation is also the keystone of tasks such as joint action tasks, in which 2 or more individuals act collectively. Joint action tasks require that the agents be both actors and observers, moving together and playing both roles simultaneously to correctly execute the action (Ferrari-Toniolo et al. 2019).

On the other hand, some studies are designed to investigate only the execution of a task in a social context, which implies the presence of others but no interaction. This is the case in enhancement and social facilitation studies (Demolliens et al. 2017), and more generally in all studies in which the influence of others on the actor monkey’s behavior is investigated. A recent body of work has focused on the modulation of the behavior of the actor based on the social consequences of the result of its actions. For example, when 2 monkeys are sitting next to each other’s and the actor’s choice can lead to a different contingency of reward delivery, the choice of the actor monkey is shaped by the future recipient of the reward (Chang et al. 2011; Noritake et al. 2018). These task designs allow the investigation of another aspect of social cognition related to the “frames of reference” in which the outcome is represented, as well as the comparison of the neurobiological substrates of outcomes that distinguish self-, other-, and both-referenced frames. We will return to this in more detail in the last section of the review.

These distinctions between active and passive task design become critical when interpreting the results of studies in the social interaction literature.

## The Neural Networks of the Self–Other Distinction

Social cognition has been defined as “the various psychological processes that enable individuals to take advantage of being part of a social group” (Frith 2008). A major role in these processes is played by different types of social signals, or information, that enable individuals to successfully interact with others. Of all the information that is acquired, the actions performed by another individual represent the most direct and observable “social cue”. Research efforts have aimed to investigate how an observer can extract information by monitoring another’s actions in a social context, such as why a specific action was performed instead of another, what its purpose was, and whether it will be repeated. Determining the neural mechanisms that underlie this general definition of “action understanding” has become one of the major challenges in the field of social neuroscience. A large amount of literature has revisited the role of the areas of the brain involved in action monitoring and understanding (Amodio and Frith 2006; Ninomiya et al. 2018), summarizing what we know about the brain regions comprising the so-called “social brain” (Brothers 1990; Frith 2007; Frith and Frith 2010). Based mainly on neuropsychological and neuroimaging studies, it has been proposed that there are 2 major neural systems in the human brain that process social information: the mirror neuron system and the mentalizing system (Van Overwalle and Baetens 2009; Catmur 2015; Ninomiya et al. 2018; Geiger et al. 2019). Electrophysiological studies of monkeys have aimed to collect evidence of the existence of such systems and their neural correlates. Monkeys are the most important animal model used by social neuroscience to explore the neural correlates underlying social functions, since they make possible a direct comparison with what we know about the same mechanisms in the human brain (Fabbri-Destro and Rizzolatti 2008; Meunier 2017).

### Two Neural Networks: Mirror and Mentalizing

The mirror neuron system was the first to be investigated. Electrophysiological studies of nonhuman primates led to the discovery of mirror neurons (MNs) in area F5 (the ventral premotor cortex [PMv]) of the macaque. These neurons increase their activity in response to both executed and observed actions (di Pellegrino et al. 1992; Rizzolatti et al. 1996), and this distinctive property has been extensively investigated. The expansive literature on this topic has led to the identification of the main brain areas of the mirror system in the macaque as the PMv and the inferior parietal lobule (Rizzolatti and Craighero 2004). Single-cell recordings in human studies suggest, however, that a wider set of brain regions may exhibit mirror properties in the human brain (Mukamel et al. 2010).

Many studies over the years have collected evidence in favor of the idea that mirror activity may play a role in how we understand actions (for reviews see Rizzolatti and Sinigaglia 2010; Casile 2013; Rizzolatti et al. 2014). Gallese et al. (1996) reported that MNs predominantly exhibit mirror activity when there is a correspondence between observed and executed actions, such as grasping or manipulating a specific object, or placing it in a specific location. Strong evidence in favor of the idea that these neurons are involved in understanding actions comes from the fact that mirror activity occurs even when the final part of the action is hidden from the observer’s view (Umiltà et al. 2001). These findings led to the idea that the activation of the mirror system in the brain could represent a bridge between the perception of an action and its understanding. The “direct match hypothesis” proposed by Rizzolatti et al. (2001) claims that visual analysis of the components of the action has to be mapped onto the motor representation of the very same action in the brain for it to be understood. There have been various hypotheses about the functional role of the mirror network, devised in an attempt to prove or disprove the idea that MNs are tied to action understanding (Hickok 2009; Ocampo and Kritikos 2011; Rizzolatti and Sinigaglia 2010; Steinhorst and Funke 2014; Thompson et al. 2019).

Regardless of this debate, it is commonly accepted that, in addition to the mirror network, another neural system is recruited during social cognition processes: the mentalizing system (Spunt and Lieberman 2013; Geiger et al. 2019). Mentalizing has been associated with the activity of specific brain areas such as the superior temporal sulcus, the medial frontal cortex (MFC) and the superior and inferior parietal lobule (precuneus, PC, and temporoparietal junction, respectively) (Amodio and Frith 2006; Van Overwalle and Baetens 2009), and it has been described as the ability to attribute mental states to others and understand others’ thoughts and points of view.

The 2 systems are thought to work together in perceiving different aspects of others’ actions during social interactions. Some functional magnetic resonance imaging (fMRI) studies (Wheatley et al. 2007; Santos et al. 2008) have investigated the level of activation of areas related to both networks in a task manipulating the perceived animacy of moving objects. They found that mentalizing areas were more active than MN areas when the moving objects were interpreted as animated. A similar pattern of greater activity in mentalizing areas than in MN areas was observed when participants were asked to understand “why” an observed action was performed rather than “how” (Spunt et al. 2010, 2011), or when subjects had to infer an emotional mood (Geiger et al. 2019). Furthermore, activation of the mentalizing system has been found to be related to the attribution of agency (Yomogida et al. 2010; Sperduti et al. 2011)—an ability that does not appear to be displayed by classic MNs (see the sections below for studies addressing this finding).

During social interaction, it is not only necessary to understand others’ actions, but also to distinguish between self and others, in order to know who is doing what. This is a distinction that canonic MNs cannot provide. It has been proposed that both networks are recruited during the understanding of actions, but that they perform different functions. When an observer observes a motor act, activation of the mirror system allows them to understand the action via a simulation mechanism based on a shared representation. When it is necessary to make an inference about the other’s mental state (e.g., when an observer needs to know why an action is performed by another individual who is perceived as animated, that is, as having a mental state different from the observer and with specific personal goals and intentions), the mentalizing system is required. The mirror system may provide a representation of a more immediate or “direct” goal, based on an understanding of a specific observed physical action, and it is thought that it sends this representation to the mentalizing system, which develops a representation of more “abstract” goals to make predictions and understand the other’s mental state and cognition (Van Overwalle and Baetens 2009; Isoda 2016; Vogeley 2017).

### The Mentalizing System in Monkeys

The discussion about nonhuman primates’ mentalizing abilities began with the seminal study by Premack and Woodruff (1978). Humans have the ability to ascribe mental states to others and to infer their intentions and beliefs, a set of cognitive skills that are collectively referred to as theory of mind (ToM). Over the years, comparative studies between humans and nonhuman primates (apes and monkeys) have sparked a lively debate about whether these “mindreading” abilities are unique to humans. Indeed, monkeys possess, or at least behave as if they possess, various cognitive skills that are usually linked to the mentalizing system: the ability to follow another’s gaze to assess their attentional state or the ability to understand what others can perceive via perspective-taking (Flombaum and Santos 2005; Canteloup et al. 2015; for a review see Meunier 2017).

A challenging debate has arisen over the possibility that one monkey can form a representation of another’s false belief (FB), which is one of the abilities typically associated with the human ToM. FB attribution is usually studied in tasks in which one monkey observes a hidden object that is moved to a different location unbeknownst to another agent. The task then tests whether the monkey correctly predicts where the agent will look for the object. Studies of macaques (Marticorena et al. 2011; Martin and Santos 2014) using a violation-of-expectation task failed to find FB attribution: the monkeys expected the agent to search in the correct location both when the agent was aware of the current position and when it was not, meaning they were not able to deduce that the agent had an FB about the location of the object. These results seem to suggest that monkeys can represent others’ mental states, such as knowledge (the agent knows where the object is because the agent has seen the object) or ignorance (the agent does not know where the object is because the agent has not seen the object), but not beliefs (the agent thinks it knows where the object is located). However, these results have been called into question recently by 2 studies (Krupenye et al. 2016; Hayashi et al. 2020) that investigated FB attribution in great apes and macaques, respectively, using an anticipatory-looking paradigm. Both species exhibit spontaneous gaze bias toward an FB target, anticipating the actions of the agent and influenced by the fact that the other agent had an FB about the location of the hidden object. More interestingly, by reversibly silencing neurons in the MFC with chemogenetic technology (DREADDs—designer receptor exclusively activated by designer drugs), Hayashi et al. (2020) found that the anticipatory gaze was suppressed, proving a causal link between the neuronal activity in this area and the attribution of an FB. Various proposals have been advanced to explain how nonhuman primates can perform tasks that require mentalizing abilities. Some researchers have challenged the definition of mindreading abilities, arguing that what might at first appear to be mental-state inference can be accounted for by a simpler “behavior-reading” mechanism; others suggest that nonhuman primates may have developed a “minimal theory of mind” (Penn and Povinelli 2007; Butterfill and Apperly 2013; for a review see Martin and Santos 2016).

Nevertheless, what matters for our purposes is that various studies agree that nonhuman primates show a remarkable ability to represent the existing relationship between information about the external world (at least that which is true for themselves) and another agent (Martin and Santos 2016) and that they can use these relationships to make correct predictions about the other’s behavior.

Although the mirror system has been widely studied using a neurophysiology approach in nonhuman primates and neuroimaging techniques in humans, the mentalizing system has been mostly investigated in human neuroimaging studies. Such studies provide insight into the activation of brain areas that are part of the mentalizing network, but they do not provide information about neural correlates at the single-neuron level.

In a recent study, single neurons were recorded from within the human dorsomedial prefrontal cortex during a classic FB task (Jamali et al. 2021). In this study subjects were required, after hearing various stories, to answer some questions that required inferring the characters’ states of mind and beliefs, and whether those beliefs were true or false. They found that neurons in this area not only selectively encoded the beliefs of others, but also distinguished between true and FBs. These results are extremely valuable because they allow investigation into the neural correlates of ToM using the most appropriate experimental paradigm; however, this can only be applied to human subjects. Furthermore, neurophysiological studies of humans are rare and limited to clinical patients. Neurophysiological studies in monkeys thus remain essential. It is for this reason that, in recent years, electrophysiological studies have attempted to investigate whether there are neural substrates in monkeys that separately represent self and others’ behavior without overlap, since this may represent the neural substrate of the mentalizing ability.

## Action Monitoring

**Figure 1:**
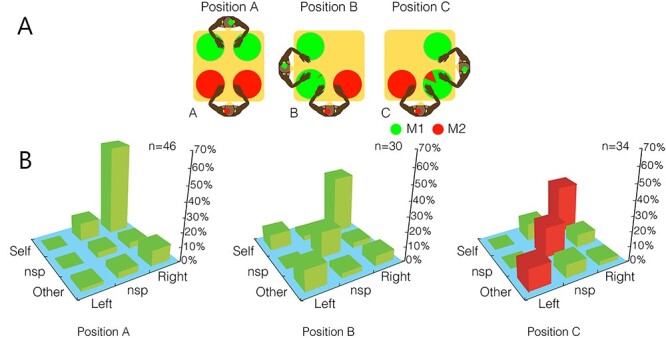
(A) Experimental conditions in the food-grab task used by Fujii et al. (2007). The circles indicate the possible locations where food could be placed on the table; colors in the circles represent the percentage of success for food retrieval for subjects M1 (green) and M2 (red). In position (A) (noncompetitive; left panel), the monkeys did not share any locations, for example, they could not reach food located in the spaces in front of the other monkey. In positions (B) and (C) (competitive; middle, and right panels), the monkeys shared one location where both of them could reach the food. (B) Percentage of neurons in M2 modulated by actor and action across the 3 task conditions. Nsp bars represent neurons modulated by motion without actor or action specificity. The proportion of other-left-responding neurons was higher in competitive position C than in the noncompetitive position (red bars indicate significantly different proportions between positions A and C). Modified from Fujii et al. (2007).

Fujii et al. (2007) began to address the question of the self–other distinction at the level of the single cell in a study where monkeys interacted by sharing a space during a food-grab task. The activity of neurons in the parietal cortex (anterior medial wall of the intraparietal sulcus; Fujii et al. 2007) was modulated mainly by self-movement toward one specific location of food on the table when the 2 monkeys were facing each other (Fig. 1A, Position A). In this noncompetitive arrangement, the food was placed on a part of the table that was accessible to only one of the two monkeys at a time. In the competitive setup (Fig. 1A, Positions B and C), the monkeys were placed next to each other, thus creating a potential conflict when the food was placed in a corner location that they both could reach. In this competitive condition, the neurons were modulated in an agent-specific manner, exhibiting a combinatory response to the movements of the self and the other and adapting their responses to this new social context (Fig. 1B).

In a subsequent study (Fujii et al. 2008), the authors identified the agent-specific properties of neurons recorded in the parietal cortex and PMv during a similar food-grab task in which the monkeys were sitting facing each other and observing various actions performed by the other monkey. Action combinations were classified based on whether self-motion (own right or left arm) or other-motion (the other’s right or left arm) occurred during a trial. In this study, the analysis of arm motion did not distinguish between reaching and grasping phases, so the responses of the motion-related neurons could have been related to either of these types of motion. The motion-related neurons in the 2 areas exhibited different properties. For example, one neuron could show a motion-related response only for other-left motions, whereas another might respond to both other- and own-left motions. Many premotor neurons responded only to the motion of the other monkey’s arm, and often with arm specificity. Although both brain areas contained a high percentage of neurons modulated by the other’s actions, premotor neurons exhibited a higher agent-specific response rate than parietal neurons. Based on these results, the authors suggested a role for the PMv in identifying the agent who is performing an action, while the parietal cortex would be more involved in the representation of the social context in which the actor is engaged, adapting the neurons’ tuning accordingly.

**Figure 2:**
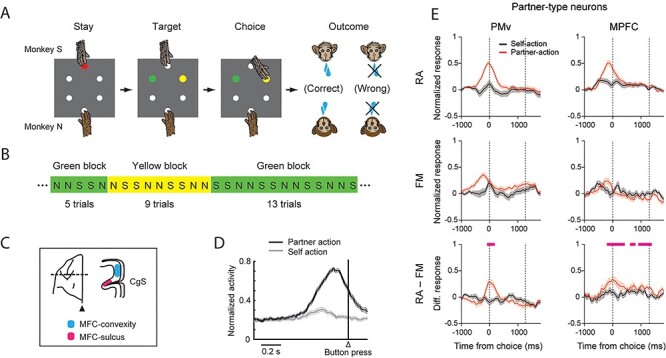
(A) Sequence of task events in the role-reversal task used by Yoshida et al. (2011). In this task, the monkeys were required to press one of 2 buttons (green or yellow) to receive a reward. Pressing one button led to reward delivery while pressing the other did not. (B) The correct response was associated with pressing a button of a specific color for blocks of a variable number of trials (5–17). Blocks could switch unpredictably, alternating between green and yellow. (C) Recording sites. (D) Example of a “partner-type” neuron, which showed a higher firing rate during actions performed by the partner than by the self. Neural activity was aligned to the button press. (E) Partner-type neuron population activity (in the PMv and the medial prefrontal cortex [MPFC]) reported in Ninomiya et al. (2020) in the animate-partner condition (RA, real agent; top panel) and the inanimate-partner condition (FM, filmed monkey; middle panel), and the difference between the conditions (bottom panel). Pink bars indicate periods during which the difference between the partner-action trials in the 2 conditions was greater than 0. Modified from Yoshida et al. (2011) and from Ninomiya et al. (2020).

The ability to monitor others’ actions for the purposes of adaptive behavioral planning has also been investigated in the motor domain in the MFC (Yoshida et al. 2011). In this experiment, the authors recorded the activity of single neurons while a pair of macaques performed a role-reversal task (Fig. 2A). The role of actor and observer alternated between the monkeys every 2 trials and the correct response (pressing a button of a specific color) switched unpredictably between blocks of trials (Fig. 2B). When the correct button was pressed, the reward was delivered to both the actor and the observer. When the actor chose the wrong button, no reward was delivered. Because the roles were reversed every 2 trials, the observer monkey had to continuously monitor the actor’s choice. Once that monkey in turn became the actor, the way it had to act depended on whether it had previously observed a success or an error. In this experiment, activity in 2 areas of the MFC was recorded: a dorsomedial region, including the supplementary motor cortex (SMA) and presupplementary motor cortex (pre-SMA) areas, and a more ventral region, including the anterior cingulate sulcus (ACCs, Fig. 2C). The authors found that 2 different populations of cells were modulated by the agent who performed the trial during the action period, termed “partner-type” and “self-type” neurons (Fig. 2D). The proportion of partner-type neurons was greater in the dorsomedial region than in the ventral part of the MFC. When the other monkey made errors, some cells selectively encoded only the partner’s errors (Yoshida et al. 2012), showing that the MFC is highly involved at different levels in the processes of self–other action differentiation.

A recent study confirmed the crucial role of the MFC in social action monitoring processes (Ninomiya et al. 2020). The authors used a role-reversal choice task similar to the one used by Yoshida et al. (2011), with the meaningful addition of a control condition comprising interactions with an inanimate agent (a filmed monkey or a filmed object). In addition, neural activity was simultaneously recorded for both MFC and PMv neurons. Partner-type neurons, that is, neurons that responded only to partner actions during the target choice period, were found in both areas, with the highest proportion in the MFC. Most of these cells exhibited a significant decrease in firing rate at the population level during partner actions when the animate partner was replaced by an inanimate filmed partner (Fig. 2E) and even more by a filmed object.

Importantly, these studies suggest that the experimental design can affect whether a correlate of the distinction between self and others’ actions is found. In fact, agent-specific responses were observed when the experimental design required an “active” interaction with an animate partner (e.g., a potential conflict situation, as in Fujii et al. 2007), or a situation requiring active monitoring of others’ choices to improve own performance (Yoshida et al. 2011; Ninomiya et al. 2020), but were largely reduced in the inanimate-partner control conditions (Ninomiya et al. 2020). When the interaction is “non-active”, as in Fujii et al. (2007), where the 2 monkeys did not compete for the food, the mutual actions lose their relevance and there is no agent-specific response. Moreover, a task designed to elicit an active interaction or a potential conflict, rather than one that requires passive monitoring of another’s actions, may more closely approximate a natural social context, which in turn could better unveil the neural correlates of this distinction.

## Predicting Actions

The ability to anticipate others’ actions is central to adapting one’s own behavior to the social context and having successful interactions. In contrast to the studies discussed above, which focused on the representation of others’ observable actions during their execution, other lines of research have investigated behavioral and neural correlates of the distinction between self and others in the domain of “action prediction”, that is, before the action becomes explicit. When we use the term “prediction” from now on, we refer to those studies that have used experimental paradigms designed to address the presence of a neural substrate that represents another’s imminent action not yet performed, that is, which predicts the other’s choices.

In this section, we examine the various paradigms used to study prediction: a prisoner’s dilemma task (Haroush and Williams 2015), an observational learning task (Grabenhorst et al. 2019), monkey–monkey interaction tasks (Yoshida et al. 2011), a human–monkey interaction task (Falcone et al. 2016, 2017; Cirillo et al. 2018), a grasping task with a human agent (Maranesi et al. 2014; Livi et al. 2019), and a computer task with a delay (Cisek and Kalaska 2004). These tasks differ with respect to the requirement to monitor the other’s actions, the type of agent involved, and the presence or absence of a delay period before the action movement period.

**Figure 3:**
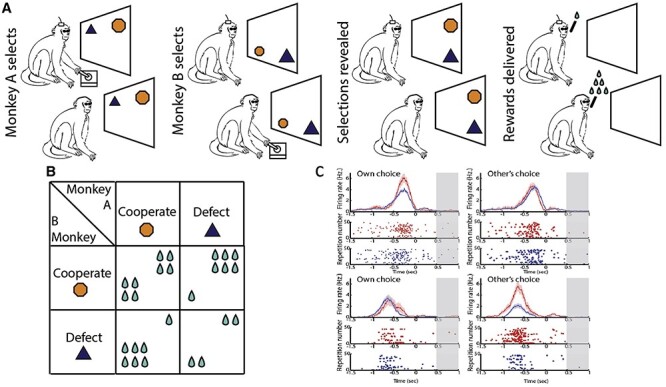
(A) The prisoner’s dilemma task used by Haroush and Williams (2015). The monkeys chose separately whether to cooperate or defect, and then their choices were shown to both monkeys and the reward was delivered accordingly. (B) Matrix of the possible outcomes based on the choice to cooperate or defect. (C) Top row: example of a neuron encoding the monkey’s own (left) but not the other’s choice (right) to cooperate or defect. Bottom row: example of a neuron encoding the other’s (left) but not the monkey’s own choice (right) to cooperate or defect. Neural activity was aligned to the monkey’s own choice before the choice of the other agent was revealed (the period indicated in gray). Red represents cooperation trials whereas blue represents defection trials. Modified from Haroush and Williams (2015).

One of the most well-used paradigms in game theory is the prisoner’s dilemma, in which 2 individuals who are separated and unable to communicate must choose between cooperation or defection. Using a behavioral task based on the prisoner’s dilemma paradigm, Haroush and Williams (2015) investigated the ability of rhesus monkeys to anticipate another’s actions (Fig. 3A). The peculiarity of this task is that the outcome of a trial depends on the combination of the individual choices of the 2 interacting partners to either cooperate or defect (Fig. 3B). The maximal individual outcome occurs when one subject defects and the other partner cooperates. However, choosing to defect could also result in the minimum outcome if the partner also defects. When both subjects cooperate, this guarantees the maximal overall outcome but not the maximal individual outcome. In this social condition, the actor monkey sat side by side with the partner monkey, where he could see it performing the task, but he could not see its choice. The actor monkeys had to predict the future behavior of the partner monkey to maximize its individual gain. The researchers found that a large population of neurons (more than a third of the task-responsive cells) in the dorsal anterior cingulate cortex (dACC) encoded the decision of the partner to cooperate or defect even before the partner’s selection was shown (Fig. 3C). The neurons involved in this type of coding were distinct from the ones involved in the monkey’s own choices. This study of Haroush and Williams (2015) thus indicated the presence of neurons specifically dedicated to the prediction of others’ actions in the dACC.

Another recent study investigated the involvement of amygdala neurons in predicting others’ choices, using an observational learning task in which 2 monkeys learned the value of specific objects and chose the one associated with the highest reward probability (Grabenhorst et al. 2019). The monkeys alternated with each other trial by trial, working on a distinct set of objects, and observational learning was encouraged by switching the objects between monkeys after a block of correctly performed trials. The amygdala neurons encoded the reward values of specific objects regardless of whether those values had been experienced or only observed. They also encoded own and others’ trials differently and, crucially, exhibited predictive activity for the upcoming choice of the partner at the time the objects were presented. The neurons that encoded the monkey’s own choice were largely distinct from those encoding the partner’s choice. Grabenhorst et al. (2019) thus hypothesized that 2 separate systems compute the monkey’s own choice and predict the other’s, possibly based on the integration of inputs received from neurons that signal value and neurons that distinguish between self and others.

**Figure 4:**
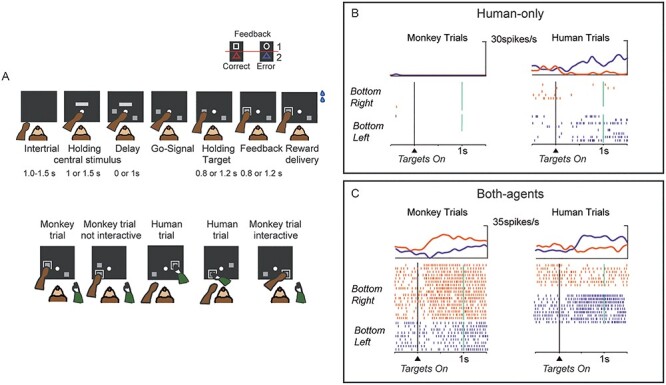
(A) Top: Sequence of task events in the spatial version of the nonmatch-to-goal task used by Falcone et al. (2017). The target stimulus represented by a gray square is presented in 2 out of 4 possible positions (top left, top right, bottom left, and bottom right). In each trial, one of the two target stimuli presented on the screen was the correct target stimulus from the previous trial, and the other was either a new one or the one not previously selected. Bottom: the monkey and the human performed the trials, switching roles. Both the human and the monkey followed the same rule: choose the target that is in a different position from that of the target chosen in the previous trial. For example, in the first human trial in the figure the human agent should not select the bottom left target, because it was chosen in the previous trial by the monkey. At the end of a trial performed by the monkey, the human agent could perform the next trial, with the monkey observing his choices and actions, or he could allow the monkey to perform another trial. (B) Example of a “Human-only” neuron exhibiting left-target selectivity only in the human trials. (C) Example of a “Both-agent” neuron exhibiting incongruent target selectivity between the monkey and human trials (right preference in monkey trials and left preference in human trials). In both (B) and (C) the neural activity is aligned to the presentation of the targets (coinciding with the beginning of the delay period). Vertical green bars represent the go signal. Modified from Falcone et al. (2017).

To ensure that the other’s behavior is under experimental control, a series of studies has used a human agent who interacts with the monkey instead of another monkey. The monkey–human interaction paradigm in a “social” variant of a nonmatch-to-goal task (NMTG) was used to investigate the activity of single neurons recorded in different frontal areas of the macaque brain. Three frontal areas were studied: lPFC (Falcone et al. 2016), the MFC (Falcone et al. 2017), and the dorsal premotor cortex (PMd; Cirillo et al. 2018). In each trial in this task, a pair of target stimuli were presented on a touchscreen. The monkeys were required to follow the NMTG rule: disregard the target selected in the previous trial and select the alternative one (Fig. 4A, upper panel). In the social variant of the task, the monkey interacted with a human partner and alternated between the roles of actor and observer during the experimental session (Fig. 4A, lower panel). In the trials performed by the human agent, the human followed the same rules. At the end of the human agent’s turn, the monkey could perform the next trial. To succeed, the monkey had to monitor and keep in memory the target stimulus chosen previously by the human partner and disregard it, choosing the alternative target, just as it would have done during sequential trials executed alone. The NMTG task was used in 3 electrophysiological studies and included a premovement delay period. Introducing a delay period allowed researchers to study the neurons involved in planning when the monkey was the actor and to identify the neural activity underlying the prediction of the choice of the human partner, rather than activity related to the mere observation of the human’s movements. During the delay period, the monkey could predict or anticipate the human’s choice because the information on which target the human should choose was available based on the monkey’s knowledge, acquired during training, that the human followed the same rules as the monkey. A large population of neurons called “agent neurons” differentiated between the agent who was performing the trial (self or other) by changing their firing rate. These neurons could also be spatially selective, thus representing the target location that the agent was going to choose. During the delay period, 3 categories of neurons were identified, based on spatial and actor selectivity. “Monkey-only” neurons showed a spatial selectivity only when the monkey was the actor. Conversely, “Human-only” neurons showed a spatial modulation only when the monkey was observing the human agent performing the task (Fig. 4B). Finally, “Both-agent” neurons showed a spatial selectivity irrespective of who performed the trial, although they did not necessarily display the same preference for target location for both agents (Fig. 4C).

A substantial percentage of Human-only neurons were found in the MFC, especially in the anterior regions (posterior medial prefrontal cortex [pmPFC] and pre-SMA), compared to the SMA, where Human-only neurons represented a smaller proportion of the total than Monkey-only neurons. Because of the delay period in the task, the participation of MFC neurons in self–other differentiation could be demonstrated, even beyond what had been shown before for the motor domain (Yoshida et al. 2011, 2012). Together, these results show that the MFC plays a major role in several stages of the process of self–other differentiation, not only during the observation of the movement of another agent, but also in the anticipation of his future choice.

The same experimental paradigm was used when recording neuronal activity in the PMd (Cirillo et al. 2018). Despite the premotor cortex being well known to be associated with mirror activity, mainly from studies targeting the PMv rather than the PMd, only a small number of cells were classified as “Both-agent” in this study.

**Figure 5:**
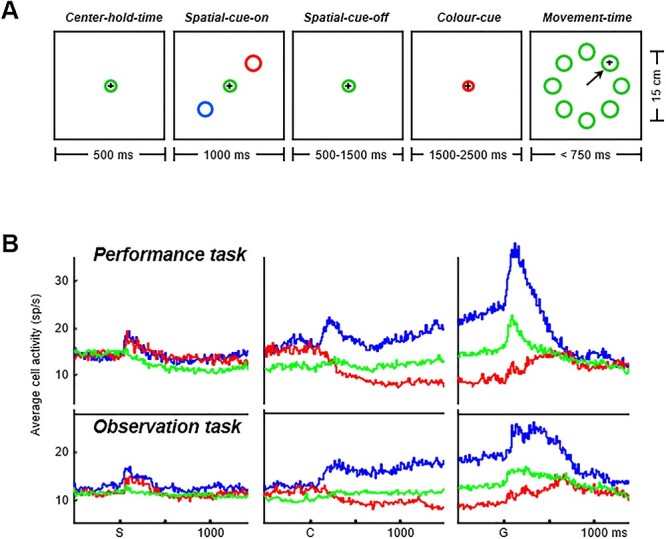
(A) Sequence of task events during the center–out reaching task used by Cisek and Kalaska (2004). Two spatial cues of different colors were placed in 2 out of 8 possible positions arranged in a circle around the center of the screen. After the disappearance of the spatial cues, a color cue of the same color as one of the two previous spatial cues was presented in the center of the screen. (B) Population activity during performance (top) and observation (bottom) conditions, aligned to the presentation of the spatial cue (S on the horizontal axis), the color cue (C on the horizontal axis), and the go signal (G on the horizontal axis). Blue traces represent trials for the preferred direction of each cell. Red and green traces represent trials in the opposite and orthogonal directions to the preferred direction, respectively. The population activity within the dorsal premotor cortex exhibited similar patterns of activation under performance and observation conditions. Modified from Cisek and Kalaska (2004).

An overlap between the encoding of own and others’ actions was observed in the PMd by Cisek and Kalaska (2004) during a delay before movement. They used a center–out reaching task to investigate how the PMd could represent the direction of reach (Fig. 5A). In that task, a color cue served as an instructional stimulus for the monkey to move a cursor on the screen with a manipulandum toward the location in which a spatial cue of the same color was presented (execution condition). In a different experimental condition, the monkeys just observed the same task sequence without intervening, while the computer moved the cursor on the screen (observation condition). They found that an overwhelming proportion (84%) of the neurons that were directionally tuned before the movement when the monkey was the agent exhibited the same spatial tuning when the monkey was the observer (Fig. 5B).

**Figure 6:**
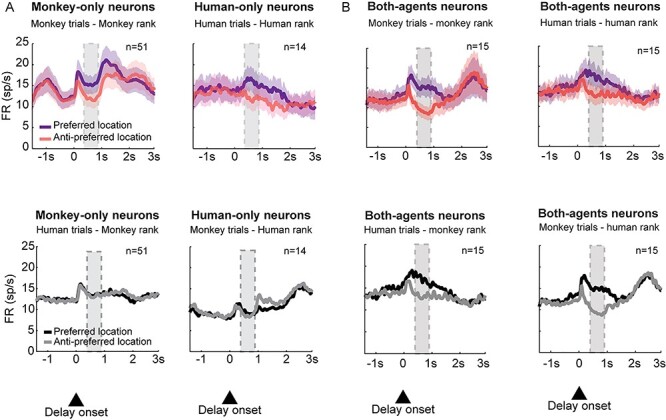
Mean population firing rate for the populations of Monkey-only, Human-only, and Both-agent neurons recorded in Cirillo et al. (2018). Neural activity is aligned to the delay onset. The gray shaded areas indicate the period of analysis (0.4–0.8 s of the delay period). Error bars indicate ± the standard error of the mean. (A) Top: Mean firing rates for Monkey-only and Human neurons in monkey and human trials, respectively. The rank that identified the preferred location was assigned to each cell individually by comparing mean firing rates in right and left trials for each agent. Bottom: Mean firing rates for Monkey-only and Human-only neurons in human and monkey trials, respectively. For each group, the activity was assigned the preferred and nonpreferred locations derived from the original trials. (B) Top: Mean firing rates for Both-agent neurons in monkey and human trials. Bottom: Mean firing rates for Both-agent neurons with the rank inverted. Monkey-only and Human-only neurons did not have the same spatial tuning in trials performed by different agents, and Both-agent neurons did not show agent-specific spatial tuning, since the majority exhibited congruent spatial preference between agents. Modified from Cirillo et al. (2018).

Although these results offer strong support for the idea that both observation and execution are represented by neurons with mirror properties, when interpreting them we should consider the specificity of the task and whether anything may have prevented self–other differentiation at the neural level. The experimental paradigm used in this study and in NMTG-based studies discussed above differ in the observed external agent (an inanimate cursor vs. a real physical agent), in the requirement for monitoring (passive observation vs. active monitoring), and also in the criterion used to test neurons during the observation condition (in the study of Cisek and Kalaska (2004) only cells that had exhibited a directional spatial tuning in the performance condition were tested during the observation condition, making it impossible to identify “observation-only” tuning). These differences may have generated a departure from “real” social interactions, promoting the activation of an underlying simulation mechanism rather than the activation of neural processes related to self–other differentiation. The interactive NMTG task promotes real rather than virtual interaction, since the 2 agents interact actively by monitoring each other’s choices. Under these specific experimental conditions at least, it seems that a minority of cells in the PMd exhibit mirror-like properties, while this region emerges as a neural substrate for the distinction between self and others. This substrate consists of 2 separate populations of neurons, which do not appear to share the same spatial preference, as opposed to those cells that are spatially modulated for both agents (Fig. 6).

### Congruent Versus Incongruent Representation

Although the neurons classified as Both-agent in the MFC might appear to be similar to the MNs in the PMv (di Pellegrino et al. 1992), because of their activation during both execution and observation of an action, it is important to note that the coding scheme of a substantial proportion of Both-agent neurons, mostly in the pmPFC, is not as fixed as would be expected if they were purely mirror. A deeper examination of the Both-agent neurons recorded in the MFC by Falcone et al. (2017) reveals a further distinction between cells that show congruent or incongruent activity depending on the identity of the actor. For example, when the monkey was the actor, some neurons showed a preference for the right target, whereas when the human was the actor, the same neurons switched to a preference for the left target (Fig. 4C). This kind of cell, which exhibited incongruent spatial activity, was still classified as Both-agent, because it was modulated by the spatial position in both monkey and human trials, even though the target preference differed. This subset of cells was relatively common in the pmPFC and rare in the SMA, pre-SMA, and PMd, suggesting a more flexible coding scheme, capable of encoding the same variable differently and reinforcing the distinction between self and others in this brain area. Lanzilotto et al. (2017) studied head movements and their neural substrates in the pmPFC and area 8 and reported a similar finding, showing cells that coded for the movements of both self and other (designated as MNs) but with unrelated preferences for the monkey’s own head movements and the other’s head movements.

The importance of distinguishing congruent from incongruent selectivity also emerged in a recent study that investigated the properties of neurons recorded in the medial parietal area V6A during an execution/observation task typically used in MN studies (Breveglieri et al. 2019). Although the majority of neurons were modulated by the type of grip only during the execution of the task, a small percentage exhibited mirror properties. Notably, these neurons were active for different types of grip in the execution and observation conditions, and therefore showed little evidence of the congruent activity typically found in classical MNs. Rather, their activity resembled the incongruent activity found in the Both-agent neurons of the pmPFC described by Falcone et al. (2017).

Recent studies on MNs have addressed questions concerning temporal activation and contributions to self–other differentiation, investigating whether this class of cells shows congruent or incongruent activity. It has been shown that in a predictable task context, when an auditory cue containing information about the action to be performed or observed is presented to monkeys, a subset of MNs in the PMv increase their activity before the actual movement (Maranesi et al. 2014). Nevertheless, in this case, the task plays a key role, leading to predictive activity even in neurons that have usually been found to be associated with movement-related modulation. In this regard, a study by Mazurek et al. (2018) reported that MN populations showed specific hidden neural states during previous and subsequent behavioral epochs, and not only during the movement period, in a reach, grasp, and manipulate task.

Later, Livi et al. (2019) used a visuomotor reaching–grasping task with execution and observation conditions to study pre-SMA neurons. They analyzed neural activity in 2 different periods of the task: the premovement period when the object was presented, and the actual movement period. Neurons were categorized depending on whether they were active only in the execution condition, only in the observation condition, or in both (mirror activity). Although neurons that were exclusively active in the observation condition were active in both periods, the majority was observed in the object presentation period, before the actual movement. Moreover, a cross-modal decoding analysis revealed that the MN coding of the type of grip during the movement period was not generalized between execution and observation, indicating incongruent activity of this type of neuron across conditions. This is further evidence of the MFC area acting as a critical node for action monitoring and self–other differentiation during social interactions. Further studies are necessary to investigate whether this particular feature of MNs in MFC can be generalized across other brain areas where mirror activity occurs. Such changes in the coding scheme at the level of single neurons do not seem to be unique to the MFC; they have also recently been shown to occur in PMd neurons during a grasping study (Papadourakis and Raos 2019).

## Outcome Monitoring

In the previous sections we addressed recent findings that reveal a separation of the representations of the actions of self and others before and during movements. In this last section, we review some recent works with similar findings regarding the representation of the outcome of actions performed by others.

Decision-making in everyday life is in part based on our ability to learn by reinforcement and associate the outcome of an action with the action itself. In this way, individuals learn how to face different situations, increasing the chances of achieving a better reward or preventing potential negative outcomes. During social interactions, such decisions are made based on the observation of others’ behaviors and by monitoring others’ outcomes. Such monitoring can also facilitate learning by observation, and it is important for extracting information from others, updating the consequences of others’ actions, and predicting others’ future behavior. Understanding the neural basis of social reward monitoring is of increasing interest to neurophysiologists who investigate the mechanisms underlying social interactions. A growing number of studies have shown the contributions of both the prefrontal cortex and subcortical structures to reward processing and social behaviors.

Among the prefrontal areas, the orbitofrontal cortex (OFC) and the ACC, which are known for encoding reward outcomes, were the first to be investigated in social tasks. These 2 cortical areas and their subdivisions exhibit distinct properties. Azzi et al. (2012) studied the OFC in a visual discrimination task in which the monkeys worked in both nonsocial and social blocks. In the nonsocial block, a monkey could earn different quantities of a reward, as indicated by a visual cue. Two other monkeys were present as observers. In the social block, one of the other 2 monkeys also received a reward. The nonsocial block was used to identify the neurons with a motivational value, for comparison with the social condition. OFC neurons modulated their activity according to both the amount of reward that was expected and whether the monkey was completing a nonsocial (it worked to receive the reward for itself only) or social task (it worked to make the reward available to both itself and the observer). Although the amount of reward delivered to the actor monkey was fixed in both blocks, the same neuron could discharge differently as a result of the subjective devaluation of the reward when it was concomitantly delivered to the observer monkey. In addition to the neurons encoding the reward value, a population of OFC neurons carried information about the identity of the recipient of the reward. Chang et al. (2013) used a reward-allocation task where the reward was not delivered concomitantly to another monkey, in contrast to Azzi et al. (2012). In this case, the reward was delivered to only the actor monkey, only the observer monkey, or to neither, following a task condition that required either an active choice or no explicit choice. OFC neurons mainly encoded the reward when it was received by the actor monkey, confirming the role of this area in the encoding of rewards in reference to self but not to others. In the same experimental paradigm, one population of neurons in the anterior cingulate gyrus (ACCg) responded specifically to the experienced or observed reward, distinguishing between the self and others, whereas another population responded to both experienced and observed reward without distinction between the self and others. The authors highlighted the fact that the neuronal responses to others’ rewards were significantly reduced in the version of the task without choice, demonstrating the importance of the ACCg in mediating vicarious reinforcement processes specifically during active choices. Furthermore, the authors also found that the majority of neurons in the ACCs were modulated by a missed reward for self.

Social reward monitoring was studied by Noritake et al. (2018) in the dorsomedial convexity region of the MPFC, corresponding to the presupplementary motor area and area 9, using a Pavlovian conditioning task for a pair of monkeys. In their task, stimuli predicting the probabilities of reward delivery for self or others were presented to 2 monkeys interacting face to face. As in the ACCg, the authors identified a higher proportion of neurons that selectively discharged in response to cues that indicated the probability of obtaining the reward exclusively for the partner. Fewer neurons responded to the presentation of cues that indicated the probability of obtaining a reward exclusively for the self. In the nonsocial controls (when the partner was absent or unable to receive the reward), the “other-type” population of neurons had a lower capacity to encode differences between the reward probabilities.

Although in this review our main focus has been on the frontal cortex, we will briefly mention some relevant work on subcortical areas and their involvement in the self–other distinction of outcomes. Several recent studies have focused on the role of subcortical structures in social reward monitoring, such as the hypothalamus (Noritake et al. 2020) and the amygdala (Chang et al. 2015; Grabenhorst et al. 2019; Dal Monte et al. 2020). In a reward-giving task, Báez-Mendoza et al. (2013) showed that a subset of neurons in the monkey striatum encoded the social agent (self or conspecific) performing an action at the time of feedback when the reward was delivered to the self, whereas a different subset of neurons encoded the agent performing the action without encoding the reward. Interestingly, these neurons lacked social properties when the conspecific partner was replaced with a computer in a control task. Also in this study, the authors investigated performance monitoring in correct and erroneous trials, and showed that striatal neurons encoded the performance error, with a small subset that was exclusively active during the errors of others (Báez-Mendoza and Schultz 2016).

Taken together, these results suggest some considerations for future work. We have examined how the social paradigm plays a crucial role in eliciting neural activity that is uniquely associated with the encoding of social information referring to others. From the studies examined, it appears that an experimental paradigm that involves only passive observation, or the observation of a passive agent, may not always be appropriate for generating representations that are specific to self and others. Thus, experimental paradigms that involve active interactions should be preferred for studying the self–other distinction. In addition to the use of nonsocial controls and active interaction, some studies have investigated the self–other distinction in terms of “reference frames” (Chang 2013, 2017). The concept of reference frames in social neurophysiology was proposed in analogy to egocentric and allocentric spatial representation observed in studies which investigated the visuomotor system (Andersen et al. 1997, Cohen and Andersen 2002). Chang proposed that socially relevant variables, such as for example the outcome of an action, may be encoded in the brain using a frame of reference that can be self or other centered, such as a spatially tuned neuron may encode the location of a specific target from an egocentric point of view (with spatial coordinates referred to the own body) or from an allocentric point of view (with spatial coordinates referred to the external world). The choice to use experimental paradigms that allow distinguishing between rewards to self, others, nobody, and combinations thereof has led to the discovery of neurons that specifically encode reward for self only when the reward is also received by others (Báez-Mendoza et al. 2013) and neurons that encode others’ rewards even as they encode a missed or received reward for self (Chang et al. 2013). The paradigms used to study the MFC can provide good examples that better explain how representations of self and others can be interpreted in different reference frames centered either on the self or on others (Chang et al. 2013; Apps et al. 2016). As suggested by Apps et al. (2016), a functional distinction can be made between 2 regions of the medial wall—the first represented by the upper and lower gyri, which correspond approximately to the dorsomedial convexity and the ACCg—and the second represented by the sulcus between them (the ACCs). Neurons in the ACCs seem to respond to others but in a self-referenced frame, that is, “*when the reward is not going to be delivered to ourselves*” (Apps et al. 2016). In the study of Chang et al. (2013), the neurons in the ACCs exhibited the same modulation for rewards delivered to others or to nobody. Thus, the task design in that study helped to show that the neural response did not refer specifically to others but rather to self, representing the reward that the individual was not going to receive. In line with this interpretation, the experimental paradigm used enabled the studies discussed above to find that neurons in the ACCs encoded the errors of others that resulted in a missed reward for the self (Yoshida et al. 2012) or the other’s future action in a task where that action was fundamental for the actor’s own success (Haroush and Williams 2015). In contrast, in the ACCg, many neurons were modulated exclusively by the other in a stricter other-referenced frame (Chang et al. 2013). This is also true for those studies that investigated self–other differentiation during action execution or monitoring and recorded neurons in the upper medial gyrus. Yoshida et al. (2011) determined that other-type neurons were more often found in the dorsomedial convexity than in the sulcus. Similarly, Falcone et al. (2017) reported a large population of other-referencing neurons in the pmPFC (areas 8–9). Based on these results, it appears that the 2 parts of the medial wall play a complementary role in social behaviors, with the ACCg specializing in encoding other-oriented information and the ACCs mediating the values of self-behavior.

## Conclusions and Future Directions

In this review, we first outlined some of the features that are important to account for to design a social task and we described the 2 main social neural systems, mirror and mentalizing. We then reviewed studies which investigated the role of several frontal areas in representing others’ actions, others’ predictive activity and others’ outcomes. In the study of action observation, attention has historically mostly been focused on the properties of MNs. However, the use of experimental paradigms that require an active (Fujii et al. 2007; Haroush and Williams 2015; Falcone et al. 2017) rather than a passive interaction, and an interaction with real (Falcone et al. 2017; Cirillo et al. 2018) instead of virtual partners (Cisek and Kalaska 2004) have enriched our understanding of the neural representations involved in social interaction. These studies have made it clearer how dedicated representations, in addition to shared representations of the self and others, can be important, and are cortically and subcortically widespread.

Moreover, these recent studies suggest that representations of actions, predictions, goals, and outcomes can be specific to others. As an analogy with the coding of spatial information that occurs in reference frames such as eye-centered or body-centered coordinates (Andersen et al. 1997, Cohen and Andersen 2002), neurons representing others can also be seen as working in reference frames, not only self-centered but also other-centered (Chang 2013, 2017). It is also emerging that, even in the presence of what may initially appear to be canonical mirror activity (i.e., an activity that does not seem to convey information about who is performing or is about to perform an action), it is possible to some extent and in specific cases to extract information concerning the self–other distinction at the neural level, especially in the MFC (Livi et al. 2019). Furthermore, neuron coding schemes can also switch, as demonstrated by Falcone et al. (2017), changing target preference depending on the actor performing the task. This is similar to what has been shown for goal coding when moving from memory to action (Marcos et al. 2019).

On a final note, further studies are necessary to investigate how neurons change their properties in different tasks, for example, when moving from tasks that promote mental simulation (with virtual agents) to tasks that elicit social prediction and introduce nonsocial controls, and to address how the neural correlates of action prediction and execution can change between multiple reference frames, both self- and other-centered, depending on the task requirements (Ray and Welsh 2018).
